# Generating New Snack Food Texture Ideas Using Sensory and Consumer Research Tools: A Case Study of the Japanese and South Korean Snack Food Markets

**DOI:** 10.3390/foods10020474

**Published:** 2021-02-22

**Authors:** Rajesh Kumar, Edgar Chambers, Delores H. Chambers, Jeehyun Lee

**Affiliations:** 1 Center for Sensory Analysis and Consumer Behavior, Kansas State University, 1310 Research Park Dr., Ice Hall, Manhattan, KS 66502, USA; krajesh@ksu.edu (R.K.); delores@ksu.edu (D.H.C.); 2 Department of Food Science and Nutrition, Pusan National University, Busan 46241, Korea; jeehyunlee@pusan.ac.kr

**Keywords:** new product development, texture, snacks, ideation, white space, marketplace

## Abstract

Food companies spend a large amount of money and time to explore markets and consumer trends for ideation. Finding new opportunities in food product development is a challenging assignment. The majority of new products launched in the market are either copies of existing concepts or line extensions. This study demonstrates how the global marketplace can be used for generating new texture concepts for snack foods. One hundred and twenty-three prepacked snack foods from South Korea (SK) and ninety-five from Japan (JP) were purchased for this study. Projective mapping (PM) was used to sort the snacks on a 2-dimensional map (texture and flavor). Sensory scientists grouped snacks on similarities and dissimilarities. PM results showed, 65% (JP) and 76% (SK) snacks were considered as hard textures, ranging from moderate to extremely hard. Sixty-five percent of JP snacks were savory, whereas 59% of SK snacks had a sweet flavor. The PM 2-dimensional map was used to find white spaces in the marketplace. Thirty-two diversified snacks from each country were screened and profiled using descriptive sensory analysis by trained panelists. Attributes such as sustained fracturability, sustained crispness, initial crispness, and fracturability were the main sensory texture characteristics of snacks. Results showed how descriptive analysis results can be used as initial sensory specifications to develop prototypes. Prototype refinement can be performed by doing multiple developmental iterations and consumer testing. The study showed how white spaces are potential opportunities where new products can be positioned to capture market space. Practical Application: The methodology produced in this study can be used by food product developers to explore new opportunities in the global marketplace.

## 1. Introduction

Food companies need to continue to innovate products to sustain market leadership. Current markets are overloaded with product offerings; thus, the challenge is to innovate new products and update existing products to gain new consumers [1]. The innovation of new products has a positive effect on the economic growth of companies [2]. Innovation helps to develop new market segments, expand current market segments and product portfolios, provide positive image building, and bring new consumers to food companies [3]. The rapid changes in technology, market trends, and consumer expectations (e.g., specific dietary, health, environmental sustainability, and packaging) is keeping the food industry under tremendous pressure to spend large amounts of money on new food product development (NPD) to either increase profits or survive [3,4,5,6].

Broadly, NPD consists of four stages, namely opportunity identification, development, optimization, and launch [1,7]. The success of NPD is directly related to several factors: (1) a unique product idea or opportunity; (2) large-scale predevelopment research; (3) superior knowledge of the market; and (4) a cross-functional team (management, scientist, marketing and launch) collaboration [2,7]. The combination of the first three factors truly determines the quality of the opportunity identification. At this stage, the idea and product developers unearth new areas of opportunities to fulfill the unmet needs of consumers [8,9]. Food companies use three primary sources for new product idea generation, i.e., the marketplace, within the company, and the environment outside the marketplace [1]. Global markets can be excellent places to explore new product ideas because those markets often provide products unknown to the developers [10].

Globalization has integrated regions, companies, markets, and societies from different countries and continents. The internationalization of markets has removed barriers for food availability and consumption and has allowed companies to explore foreign markets for product innovation and idea generation [10]. Food companies have successfully developed global food products by generating ideas and products and one country and moving those ideas or products to other countries; for example, beverages (e.g., Coca-Cola and Pepsi), tea (e.g., Lipton), coffee (e.g., Nescafe), cigarettes (e.g., Marlboro), or chewing gums (e.g., Wrigley). The inclusion of international markets in NPD for generating new product opportunities offers a great diversity of products, customers, and consumers. Food companies use data (consumer involvement, food trends, and environmental factors) most frequently in the opportunity identification and product design stage of NPD [11]. Thus, researchers and food companies need to find both novel and quality opportunities from the market [12]. These gaps (white spaces) could be potential unmet consumer needs that can be filled by developing products for these identified consumer needs [12].

The main task of NPD is to develop products that deliver desired benefits to their intended consumers. Developing consumer-centric products involves great risks and failures [2,3,13]. Fuller [1] identified two main early-stage risk components in NPD: (a) wrong investments in new products that would later fail in the market, and (b) overlooking a potentially successful new product, termed an opportunity loss. Dijksterhuis [14] explained five factors for a high number of new product failures: (1) the uncoordinated efforts of many different functions working on different aspects of consumer and product development; (2) lack of understanding of consumer behavior; (3) usage of outdated research models; (4) lack in seriousness towards behavioral sciences; and (5) high reliability on the notion that good-quality products automatically lead to high sales. Even after producing a large amount of literature on NPD, the failure rate is still very high. Between 2011 and 2013, 76% of the newly launched consumer goods did not survive one year on the market [15], 45% of products remained on the market for less than half a year [14], 75 to 95% of newly developed food and beverage products failed within one year of launch [16].

To increase the odds of NPD success, many researchers recognized the need to consider consumer behavior and choice-based ideas from external global markets [9,17,18,19,20,21,22]. Sensory science and consumer research provide techniques to identify white spaces in NPD, support research and development, and contribute to minimizing the decision uncertainty [23].

Researchers have identified the early stages in NPD as the most important activities for both product success and failure [1,24]. The early stages of NPD have sometimes been termed as the “fuzzy front-end” because they are looking to take vague ideas and provide some clarity in understanding actual needs. Unfortunately, they also have been called “fuzzy” for reasons such as ill-defined processes, ambiguities, confusion, and ad hoc decisions [18,24]. The early involvement of sensory and consumer research in NPD is recommended as an important success factor [2,23,25,26]. Thus, there is a need for a structured sensory science-based framework in the early stages of NPD for idea generation [3]. The use of techniques such as interviews, focus groups, behavioral observation, ethnography and other such qualitative measures plays an important part of the process for determining and documenting consumer needs [27,28,29,30,31,32,33]. In addition, quantitative measures of consumer understanding, attitudes, behaviors, and emotions as they relate to products provide additional information that may be critical to discerning potential product requirements [34,35,36,37,38].

A sensory method called projective mapping (PM) or “napping” is used as a tool to categorize products and discover white spaces among product groups. In PM, assessors position the products (samples) on a two-dimensional space according to the similarities and differences of product characteristics [5,39,40,41,42]. PM has been described as a natural, holistic, and spontaneous way for people to describe products. It has been successfully applied to various food products, e.g., orange juice [43], red sufu [44], wine [45], pork [46], peas, and sweetcorn [47]. The influence of extrinsic factors on a consumer’s perception of foods such as smoked bacon [48], fermented dairy products [49], and chicken meat [50] as well as packaging [51] also has been studied with PM. Over the years, PM or “napping” has been shown to be efficient, timely, and cost-effective, to obtain a “big picture” overview of a category and is considered a rapid method for gauging some descriptive sensory attributes. The application of PM as a sensory tool for rapid product categorization and characterization for a large number of products is common [52].

The early stage of NPD includes brainstorming and ideation by looking at consumer and market trends. To develop new concepts, researchers and food companies obtain information from competitive food products in the market and then develop concepts for new products. Using descriptive sensory analysis gives an edge to the researcher in a better understanding of competitive products, and of the marketplace where the potential new product will be placed [53]. Descriptive sensory analysis is a classic sensory method used in NPD to profile products on all of its perceived sensory properties [54,55]. It involves the discrimination and description of both quantitative and qualitative sensory attributes by trained sensory panelists [53]. The descriptive analysis offers various applications such as help in understanding the relationship between sensory and instrumental measurements, the relationship between descriptive sensory and consumer preference measurements, product optimization and validation, product profiling, quality control (product comparison), sensory mapping and product matching, shelf life and packaging effect, etc. [53,56,57,58,59,60].

The descriptive profiling of foods helps to identify the main sensory attributes of food products which can be manipulated: (a) to create a profile of desirable sensory characteristics to help in the development and (b) to define early-stage specifications for a new product [53]. The key sensory attributes that are identified help to distinguish the importance of “tangible” product characteristics that form the basis of technical product specifications [18,25]. Sensory characteristics are measurable and can be manipulated, and therefore, characteristics obtained from a wide range of products can encourage the researcher to create a product with different and multiple sensory profiles [53,61]. Descriptive profiling methods have been used to profile many products including products such as bread [62], fresh and dried mushrooms [63] snacks and snack-like foods [64], potato varieties [65], mate tea [66], ground beef [67], and smoked food products [68]. Many sensory studies combined descriptive analysis results with consumer hedonics to determine why food products are liked by consumers [25,69,70]. The combination also helps to identify consumer segments and their specific sensory preferences for certain product characteristics, and also give insight into possible gaps in the marketplace [71,72].

Consumers describe a product’s benefits by perceived intrinsic and extrinsic characteristics (e.g., the crispiness of potato chips [73], creaminess in dairy products [74,75], “health, good taste and convenience” [17]. Principal components analysis (PCA) plots generated on descriptive sensory profiling data provide an opportunity to access the positioning and comparison of products in the market space [53]. Using PCA plots, several white spaces (the open space between products) and product clusters can be identified with their identifying main sensory attributes [53]. Those sensory attributes are reported to be directly experienced by consumers to assess products’ evaluation and significantly influence consumer product appraisal [76]. The “white spaces” suggest areas where new products could be developed to meet unmet needs [10,77,78,79]. However, the presence of white space does not necessarily mean that (a) products do not exist in that space but only that they were not part of the study, (b) just because a product is made to fill the space that the product will succeed, or (c) it is impossible to develop a product that fits the white space based on current technology.

A goal of this project was to highlight one strategic framework to find white spaces in the marketplace and then develop new snack texture concepts to fit the sensory concepts identified as white spaces. The specific objectives were to (a) find the new texture and flavor gaps in several large-scale markets; (b) identify key sensory texture characteristics of the Japan (JP) and South Korea (SK) snack foods; and (c) to demonstrate how unfamiliar marketplaces can be used in NPD for ideation. This study is a continuation and expansion of earlier work [10].

## 2. Materials and Methods

### 2.1. Materials

One hundred and twenty-three packaged snacks from Seoul and Busan, SK, and ninety-five packaged snacks from Kyoto, JP, were purchased in-country and shipped to the Center for Sensory Analysis and Consumer Behavior, Kansas State University (KSU), United States (US). Although a wide range of products other than those thought of as traditional snack foods are eaten as snacks [80], fresh fruits, candies and confectionary products often eaten as desserts were excluded from this study to focus on foods that were made and marketed primarily as snack foods. Trained sensory scientists and product developers from the US, China, India, and SK purchased snacks for this study, following a product procurement strategy recommended by Murley [10] to help ensure that the wide range of snack foods and types was represented. Package guidelines were followed for storage and handling.

### 2.2. Snacks Data Bank

Information related to each snack type such as product name, product description, manufacturer, package size, number of packages, ingredient list, and pictures (front and back) were collected to develop a snack data bank for each country (See the JapaneseSnacksDataBank.xlsx and SouthKoreanSnacksDataBank.xlsx at https://krex.k-state.edu/dspace/handle/2097/40897) (accessed on 2 January 2021). The collected data helped in product identification, product cataloging, and most importantly in knowledge generation about various snack foods such as packaging data, as well as the ingredient and nutritional data. Several authors concluded that knowledge generation on market products, and its proper integration with organizational learning are important aspects of NPD [3,13].

### 2.3. Projective Mapping

PM was used in its original concept as described by its authors with few modifications [42,81]. A subset of purchased snacks with the most diversified texture profile, new ingredients, and novel concepts were selected from each country for PM. Fifty-one snacks from JP and sixty-six from SK were included in the PM. The modalities used for PM were texture (hard to soft texture perception) and flavor (savory to sweet flavor perception). Snack foods were sorted for similarities and dissimilarities on the aforementioned modalities. The panel determined the key aspects for placement. The snacks were tasted blind with only a two-digit code and sorted into groups by six trained sensory scientists with prior experience in snack food evaluation.

**Figure 1:**
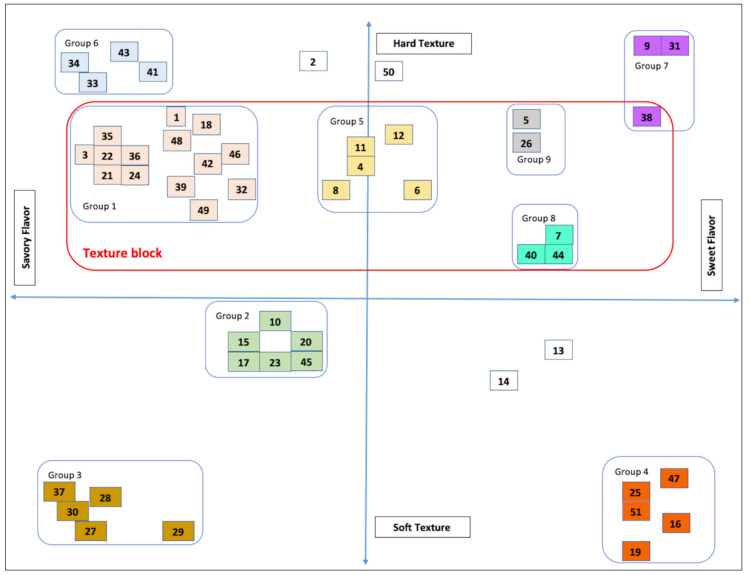
Projective mapping plot of the fifty-one JP snacks showing nine product groupings and outlying products (snacks are coded with 2-digit numbers and snacks with the same color are in the same group). The products’ grouping was subjective.

Two 1 h training sessions were held to orient the panelists with products; training included tasting samples. PM was performed on a rectangular des covered k; the center of the desk was labelled for axis interaction and extreme ends were labeled exactly the same as represented in Figure 1. Panelists evaluated one sample at a time, discussed and reached consensus on positioning the samples. The number of samples evaluated in each session was restricted to ten samples. Additional sessions were held after a wait of at least 1 h. When all the samples for a country had been tested, discussed, and placed on the desktop, the panelists reviewed the placement and made any final modifications. At that point, the “x” and “y” coordinates of the desk were measured to provide the specific data for each sample. Water and unsalted crackers were used as palate cleansers. The products were grouped subjectively (based on the perceived texture and flavor evaluation).

### 2.4. Snacks Sensory Description

After PM the entire set of products and examining the results, 20 snacks from each country were selected to represent the entire map and were screened for descriptive sensory profiling. To increase the product pool size, 12 new snack products from each country were also added for descriptive profiling. The parameters used to screen snacks included the coverage of the map surface and the selection of diversified and novel textures, new ingredients, and novel concepts. The screened snack foods are listed in Table 1 (for JP) and Table 2 (for SK). In addition, three snacks (Stacy’s pita original, Lay’s classic potato chips and Tostitos original corn chips) widely available around the world also were included in the test to provide a “reference” set of products that could help anchor the maps. This also allows other researchers to help better understand the similarities and differences shown on the map, particularly because many would never have seen or tasted the products tested.

### 2.5. Descriptive Profiling

Consensus methodology was used to develop sensory attributes, definitions, and references [55,82]. Panelists and the sensory analysts determined attributes for further rating by consensus. The final list of attributes was kept consistent for both JP and SK snacks. The snacks were profiled for flavor, amplitude, appearance, and texture attributes. However, because the flavors of many snack foods can be easily changed based on consumer preferences and many of the snacks tested come in many different flavors, only appearance and texture attribute data were considered in this analysis and are shown in this paper. The texture terms used in descriptive profiling were adopted from the snacks texture lexicon published by Kumar and Chambers [64].

Panelists used a scale ranging from 0 to 15.0 with 0.5 increments where 0 represents none and 15 extremely strong to profile snack samples. Each panelist independently allocated intensities to the attributes and then the intensities were discussed within the panel to reach a single consensus score for each attribute for each product. Three samples were evaluated in each session. Panelists cleaned their palates between samples with freshly cut cucumbers, mozzarella cheese (manufactured by Kroger, Cincinnati, OH, USA), hot water, and a washcloth for the cleaning of lips and hands. The descriptors list, definitions and reference standards are provided in Supplementary Table S1. Similar methodology has been used in other recent studies for the sensory profiling of various foods, e.g., [62,63,64,65,66,82,83].

### 2.6. Sample Preparation

The snacks used were all ready to eat and needed no preparation; they were served as they were. The samples were blind coded with three-digit codes, served in 8 oz (Styrofoam) and 3.25 oz (plastic) cups (based on the size and shape of the snacks) covered with a lid. One sample at a time was served to panelists in a randomized order.

### 2.7. Panelists

For the PM, six sensory analysts with experience in snack food evaluation served as the panel for the study. All of the analysts had training in PM techniques and worked as a group to produce a single joint map of snacks for each country. The panelists were trained to specifically focus on the texture and flavor stimuli. The assessors were tasked to screen the large pool of samples, position them on a 2-dimensional space by reaching a consensus on the general differences on texture perception. The objective of PM using a trained panelist was to layout an overall product space rather than generate data through scaling differences. After PM, the descriptive study was planned to identify the subtle difference and quantify descriptors among different panelists.

For the descriptive analysis, six highly trained descriptive sensory panelists were used for this study. Each panelist had more than 120 h of training in descriptive panel training and more than 1000 h of descriptive testing experience with various types of foods and beverages, including extensive testing on different snack type products. The panelist worked on evaluation techniques for appearance, texture, and flavor perception. The panelist received 9 h of additional orientation with both the JP and the SK snacks. The number of highly trained panelists who participated in this study was sufficient to differentiate the samples in the descriptive analysis [84,85,86,87] and similar panels have been used in other studies [66,88,89,90,91,92].

### 2.8. Data Analysis

Correlation-based principal component analysis (PCA) and agglomerative hierarchical clustering (AHC) were performed on the sensory descriptive data using data analysis software XLSTAT 2019.3.2.61545. To prevent data redundancy, attribute correlations were analyzed by the data analytical software R-studio version 4.0.0 (R Foundation for Statistical Computing, Vienna, Austria; https://www.R-project.org/) (accessed on 10 January 2020). Note that for consensus profiling, because there is no variance in scores, “significant” differences are not determined [55,82]. Instead, the size of intensity differences deemed “important” is determined in advance by researchers. In this case, differences were deemed important if they varied by ≥0.5 points, a typical level used in such studies.

## 3. Results

The sequential use of sensory tools produced information on the main sensory descriptors, the snacks market categorization based on sensory descriptors, existing snacks market space, and white spaces (potential opportunities). All that information was produced by the PM plots and subsequent PCA mapping along with the original data. The information can be used by a snack manufacturer to (a) have an overview of the snack markets (based on sensory parameters); (b) identify the major flavors, textures, and possible trends; (c) learn about a competitor’s product positioning; (d) develop new concepts to bring to further sensory (including consumer) research; and (e) enhance their product snack portfolio. The results explain how this information can be generated using JP and SK snacks as examples.

### 3.1. Projective Mapping

**Figure 2:**
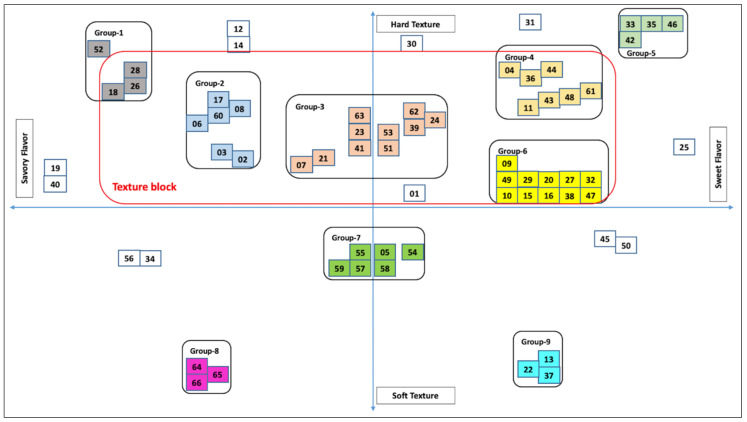
Projective mapping plot of the sixty-six SK snacks showing nine product groupings and outlying products (snacks are coded with 2-digit numbers and snacks with the same color are in the same group). The products’ grouping was subjective.

The representative maps of the PM results are presented in Figure 1 (for JP) and Figure 2 (for SK). The snacks are coded with two-digit numbers for representation purposes.

#### 3.1.1. Japanese Snacks

Fifty-one snacks with a variety of texture profiles were sorted into nine groups (Figure 1). The PM was primarily focused on the textural dimension from a hard to a soft texture. Because the snacks were seasoned with different types of flavors, sorting them based on flavor was much too difficult for a 2-dimensional space. The only flavor dimension that was considered was that from savory to sweet. All the products were analyzed visually, in the hand (tactile hand feel), and tested orally (for texture and flavor) by the sensory scientists who participated in the PM.

Out of 51 snacks, 33 snacks (64.71%) were considered as hard bite textures, ranging from moderately to extremely hard. The main texture descriptors were crispiness, crunchiness, sustained crispiness, sustained crunchiness, and hardness. The largest snacks group (group-1) had 14 products (for example, crackers, wafers, puffs, and rolls), representing 27.45% of total snacks. Similarly, group-6 had four snacks, grouped for extremely hard texture and strong savory flavor. Group-2 had six snacks (for example, corn trumpets, corn puffs, squid crackers, shrimp crackers, cheese-filled sticks, and unbranded grain crackers), representing a soft-bite texture with of the mild savory flavor category. A complete list of the JP snack food groups is presented in Table 3.

Group-1 represents the largest portion of JP snack foods from the selected snack pool. The results suggest that most JP snack foods are hard to bite texture snacks seasoned with various flavors such as savory, bland, and plain salt. Group-1 and -5 differed in terms of flavor intensities but were similar on textural dimensions. Collectively, snacks from groups-1, -5,-7,-8, and -9 formed a large hard texture block (highlighted with a red border) (Figure 1). The hard texture block accounted for 49% of the overall JP snacks market space. Hard texture snacks appeared to dominate the JP snacks market, which has a large number of existing products. The possible explanations could be (a) JP consumers prefer hard texture (crunchy and crispy) snacks; (b) our research team inadvertently collected more hard texture snacks and therefore limited the product pool; or (c) it is a true representative of the JP snacks market. Hence, for a new product developer, understanding the texture dimensions of JP snacks could be a potential framework or area of interest to explore either as copycat products (harder textures) or to create new textures (e.g., at the softer texture end of the spectrum). Of course, another niche area could be bringing new flavors into the existing texture spectrum where flavors may be lacking.

Thirty-three snacks (65%) were savory, including snacks seasoned only with plain salt. Other flavors (for example, seafood, seaweed, prawns, squid, crab, and fish) also were present in that grouping. Savory flavored snacks occupy the largest space in the JP snack market. Thus, for a product developer, a savory flavor could be an easy carry-over from one snack type to another, but also positions the product against a larger competitive set.

The broad range of textures and flavor, some of which were not found in tests conducted on snacks from other countries represent a new opportunity for manufacturers to transfer ideas from one country and culture to another. Taking ideas for new products from countries with a plethora of products often is an easy way to create new products for countries where existing products may be in more limited supply or exist in fewer sensory segments.

The gaps between the product grouping are the white spaces where no products were found to exist. Those empty spaces are potentially unexplored opportunities in the JP snack market and perhaps in other markets. The bottom half of the plot in Figure 1 represents the soft texture snacks space. More white space is available in soft texture snacks over hard texture snacks. This may be because (a) a smaller number of products are in the soft texture product pool (a potential opportunity), or (b) the JP consumer does not prefer soft texture snacks. If soft texture snacks are not as popular in various countries, they may not be a real opportunity. For JP, the further investigation of that snack segment is required in terms of consumer studies. For other countries, the opportunity for new snack development in the sweet category needs to be considered and further research with potential new products may be warranted. In addition, spaces that are not filled with many products also may be considered “white” spaces. For example, the space between group-1 and group-7 has only five products (i.e., group-5). Considering the number of products that exist in other areas of the map, more products could be developed to fill and position in this space.

The plot can be divided into four quadrants (Figure 1). The first quadrant (Q1) represents hard texture snacks with a sweet flavor, the second quadrant (Q2) is hard texture snacks with a savory flavor, the third quadrant (Q3) is soft texture snacks with savory flavor, and the fourth quadrant (Q4) is soft texture snacks with sweet flavor (Figure 1). Each quadrant produces different information. For example, Q4 and Q1 have the least number of snacks and more white spaces. A product developer can develop a wide range of new textures (hard to soft) with sweet flavors. The market space offered in these two quadrants is quite large. Similarly, other quadrants can be used to frame initial product concepts, either individually or in combination with other quadrants.

From a broader perspective, the plot can be divided into two halves. If a product developer is interested in new snack flavors, they can divide the plot on the vertical axis (Figure 1). For example, the left half of this plot, vertically divided, characterizes the savory flavor market space ranging from a hard to a soft texture. The right half of the plot represents the sweeter flavors market space with the same texture range from hard to soft. If the plot is divided into two halves on the horizontal axis, the top half contains all hard texture snacks with both sweet and savory flavors. The bottom half of the plot comprises all softer texture snacks spreading across savory and sweet flavor. There is a wide range of options that could be explored in soft texture with savory flavors. For example, there was no “soft texture, non-seafood” savory snack found in this study. Only 18 snacks were of soft texture, mainly groups-3 and -4. Group-3 consists of fish or seafood flavored soft chewy snack loaded with strong sour-savory flavors. In addition, group-4 snacks were soft textured sweet snacks but not chewy. Considerable white space is available across the savory-sweet flavor dimension with a soft texture profile that may help the developer in identifying additional products for the market.

One issue that must be considered is that many softer textured snacks were found when conducting the initial product search. However, many of those were in the form of freshly prepared “street snacks”, such as fresh seafood or egg products that could not be sold in a shelf-stable manner given current technologies. Those products may be considered as inspiration for manufactured shelf-stable products but also represent a competitor that is not directly accounted for in this research.

#### 3.1.2. South Korean Snacks

A total of sixty-six pre-packed snacks were sorted between the texture (hard to soft) and flavor dimensions (savory to sweet). Nine main groups were formed (Figure 2). Group-6 had eleven moderately hard texture snacks with a mild sweet flavor, group-3 had ten moderate hard texture snacks with a bland flavor, group-4 had seven sweet snacks with slightly harder texture than groups-6 and -3. Group-1 had four extremely hard texture snacks with an extremely strong savory flavor, and group-5 snacks had a similar texture but strong sweet flavor. Group-2 snack texture was similar to that of group-3. Group-7 snacks had bland flavors with a slightly softer texture compared to group-3. The other two groups representing soft texture snacks were groups-8 and -9. Both groups were similar in the texture dimensions, with group-8 snacks being savory and group-9 being sweet. A complete description of the groups, texture, flavor, and snack names are provided in Table 4.

Group-3 snacks were bland or seasoned with plain salt. Group-7 snacks were seaweed flavored with a slightly soft texture. Overall, 12 snacks, mainly from groups-1 and -2 were seaweed flavored. Group-8 snacks were savory chewy meat/seafood snacks. Group-9 snacks were savory with a soft texture. Thirty-nine (59%) snacks were sweet-flavored or lingered with a sweet taste. Among sweet-flavored snacks, thirty-one (47%) had a slight to moderate hard texture and only eight snacks were soft textured. The PM results obtained from the pooled products showed that the SK market had more sweet snacks over savory.

PM results showed that fifty (75.8%) snacks were in the hard-textured space, varying from slightly hard to moderately hard. Only nine snacks were of extremely hard texture. PM results indicate that the SK snack market space is mainly constituted of slightly to moderately hard texture snacks. The texture dimensions of the SK snacks market were similar to the JP snacks market but with slightly less hard textures. The white space in soft texture products either with savory or sweet flavor is due to the small number of snacks available in that segment. Overall, slight to moderate hard texture with low-intensity sweet flavor can be said to be the best description of the SK snack market. The texture dimension of SK snacks mainly varied from moderately hard to slightly hard with most being sweet flavored. On the other hand, the texture dimension of JP snacks varied from moderately hard to extremely hard and seasoned with savory flavors.

The PM results helped to identify the existing snack food positioning in the market space. This enabled researchers to do a product segmentation and explore white spaces for new opportunities. The developers can look at PM plots as a whole, or as individual quadrants, or half plots to find new product opportunities.

### 3.2. Descriptive Profiling

#### 3.2.1. Japanese Snacks

**Figure 3:**
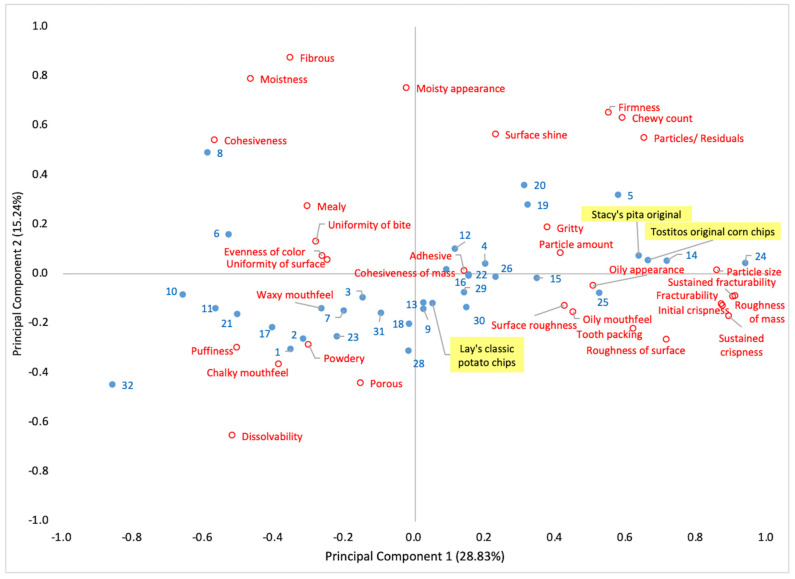
Principal Componensts Analysis (PCA) plot representing the descriptive texture profiling results of JP snacks. The numbers (including dots) highlighted in blue color represent the snack type as listed in Table 1, and the text (including dots) in red color denotes texture attributes. Three US snacks—Stacy’s pita original, Lay’s classic potato chips, and Tostitos original corn chips (highlighted in yellow color)—were used to compare texture dimensions with JP snacks.

Thirty-three texture descriptors were used to profile thirty-five snacks. The PCA plot obtained from the descriptive data is presented in Figure 3. The product variability explained by the first two principal components (PCs) was 44.07% of the total variability. The main differentiating texture attributes were PC1 (initial crispness, fracturability, roughness of mass, sustained fracturability, sustained crispness, cohesiveness, dissolvability, puffiness and firmness) and PC2 (dissolvability, surface shine, porous, cohesiveness, surface roughness, roughness of surface, and puffiness). One set of snacks featured high-intensity scores of PC1 attributes, the other set of products highlighted strong intensities of dissolvability, powdery, porous, and chalky mouthfeel. Another large set of snacks close to the center of the PCA plot represented low intensities of attributes such as adhesive, cohesive of mass, waxy mouthfeel, gritty, mealy, uniformity of bite, and uniformity of surface.

The PCA plot provided a space where new products of certain textures could be developed. For example, there is a scarcity of snacks that are fibrous, cohesive, mealy, moist, having waxy mouthfeel, etc. Similarly, a large white space can be seen around descriptors such as firmness, chew count, gritty, etc. The developer can utilize descriptive data to incubate new texture profiles to fulfill empty texture spaces by introducing new prototypes. The analytical descriptive profiling data can be used as a reference guide to shape new prototypes for further development [24,56]. Of course, white spaces such as the one mentioned in the firm, chewy, gritty area may be undeveloped because that product “concept” may not be appetizing for consumers. However, some products, such as meat jerky, may fit with some aspects of that concept. We also imagine that some high protein products made from plants might fall into that category and whether they are successful or not may depend on accentuating characteristics that might be desirable (firm, chewy) in certain contexts, while reducing characteristics that usually are less desirable (e.g., gritty). Overall, the descriptive sensory profiling can help to design the prototype, determine prototype requirements, and define the key sensory specifications [18].

#### 3.2.2. South Korean Snacks

**Figure 4:**
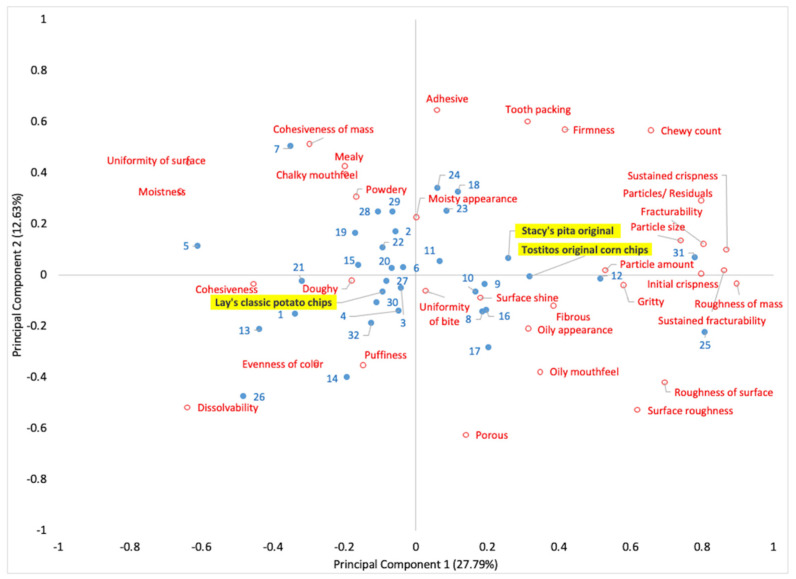
PCA plot representing the descriptive texture profiling results of SK snacks. The numbers (including dots) highlighted with blue color represent the snack type as listed in Table 2, and the text (including dots) in red color denotes texture attributes. Three US snacks—Stacy’s pita original, Lay’s classic potato chips, and Tostitos original corn chips (highlighted in yellow color)—were used to compare texture dimensions with SK snacks.

The PCA plot representing texture descriptive results is shown in Figure 4, with three main snack clusters being noted. The largest groups of snacks had moderate intensities mainly described by the cohesiveness of mass, uniformity of the surface, mealy, chalky mouthfeel, moistness, and adhesive. The second group of snacks was profiled by cohesiveness, doughy, evenness of color, puffiness, and dissolvability. The third group of snacks with strong intensities of texture attributes was marked by PC1. The snacks with strong intensities are represented on the edges of the PCA plot, whereas the snacks with low intensities of textures attributes are located near the center of the PCA plot (Figure 4). The first (PC1) and second principal (PC2) components explained 40.42% of the total variability. The texture attributes contributing to PC1 were dissolvability, cohesiveness, roughness of mass, initial crispness, fracturability, sustained crispness, and roughness of surface. The texture attributes for PC2 were roughness of surface, dissolvability, firmness, fracturability, and initial crispness.

Large white spaces between and within snack groups are present. For example, the white space around Stacy’s pita original chips shows the unavailability of a similar product in the SK snack market. Similarly, white space around the Peacock Florentin Coconut French dessert, prawn snack, and Heyroo noodle snack shows where new texture concepts could be developed to fill these spaces. The developers can use the tested products as references to quantify texture descriptors.

## 4. Discussion

This research work adopted a market assessment and product category appraisal approach for new product ideation [90]. This research work applied sensory tools to deliver a pool of new texture concepts. The developer can narrow down the list of new concepts after evaluating consumer response and technical feasibility. The discussion below explains how a step-by-step process can be used to funnel new ideas.

Step 1: Pre-development Homework (Preliminary Market Assessment, Which Markets and Why?)

Detailed preliminary homework was conducted to explore the JP snack market [10], and similar work was done for the SK market, except that an in-country sensory professional was used to help the process move more quickly. The critical sections covered in the pre-development homework includes an assessment of the JP snack market potential, desired snacks market portfolio, the size, feasibility, and area of interest. The other pertinent segments were market selection, location, information acquisition, innovation trends, funds, skilled teams (manpower), product procurement strategy, product shipment, timelines, climate, travel, lodging, boarding, storage, and shipment, etc. Pre-development work is considered important in NPD [3,10,13,18]. During the early stages of NPD, researchers aim to search for novel ideas (for example, texture, ingredient, shape, size, packaging, convenience, and flavor) [1,93,94]. Many researchers reasoned that earlier stage work such as market exploration is most beneficial for the NPD process [1,11,95].

Step 2: Market-Driven Product Assessment

A deep understating of the nature of the market, competitive index, and consumer trends are essential for new product ideation and success [96]. Failure to understand market orientation, assessment, and leaving consumers out of the development process could lead to disasters for innovators. The notion of deep market research to discover white space is supported in several studies [12,18,78]. Researchers undertook a detailed market assessment of the markets which included the participation of local consumers from both countries. A multi-stage market assessment process includes different teams exploring different zones of the market, product procurement strategy, consumer interviews, daily sensory evaluation by sensory scientists, information collection, and shipping enough quantities from the market for further investigation [10].

Once snacks were procured, sensory tools such as a 2-dimensional PM were applied to sort the products into groups. The snacks were segmented for texture and flavor modalities. Sixty-five percent of JP snacks had hard textures (ranging from extremely hard to moderately hard). Results indicate that a big block of snacks across the flavor dimension accounted for 49% of the snacks market space. PM results are a close representation of the JP snacks market space.

The PM tool helped to portray each country’s existing snack market texture and flavor outlooks. PM enables the researcher to perform a product segmentation and explore the white spaces in the market. New ideas can fill the white spaces by testing with consumers through models, mock-ups, product concepts, and actual prototypes [18,19,97]. Once the new product concepts are extracted, they should be tested to explore insights on consumer relevance [24]. The initial inputs from the consumers on the needs, likings, and preferences can help to screen and envisage these concepts. A thorough market assessment is a key step in NPD [1,96]. Developers also can use any other sensory dimensions to sort products based on their interests. For example, scientists who work on product renovation or novel ingredients can also use PM as a tool to identify an ingredient’s market space.

In the SK snacks, PM results showed that 75% of snacks are hard textured, varying from slightly hard to moderately hard. Fifty-nine percent of SK snacks are sweet flavored or had a sweet aftertaste. Among the sweet-flavored snacks, 47% were hard textured and only eight snacks were soft textured. PM results obtained from pooled products show that SK consumers eat more sweet-flavored snacks than savory.

The overwhelming presence of hard bite texture snacks in the JP and SK market also reflects the product characteristics that currently drive consumer interest. This also advances the need to explore detailed texture attributes that form a product profile. Once foundational characteristics such as the hard bite texture are framed, then the developer seek to measure these texture attributes via descriptive analysis. By identifying what texture attributes form product characteristics (for example crispness, fracturability, firmness in case of snacks) the developers can cement inputs for the subsequent technical prototype developmental stage [2,8,9,26,98].

Large white spaces were discovered on the soft texture axis for both countries. In addition, considerable white space is available across the savory–sweet flavor dimension with a soft texture profile in the JP market (Figure 1). This may be related to the lack of creative product development and marketing in that space, lack of consumer interest in those textures or flavors, or a lack of technology to satisfactorily produce such products. We believe that based on the products encountered, the white space likely results from a lack of product development in that space and the lack of technology to produce products suitable for that space. A number of freshly prepared “street snacks” with a softer, sweeter profile were available in JP, but those products would be difficult or impossible to reproduce today because of distribution and shelf-life issues. For example, a seemingly popular snack of prepared seafood and egg that was both sweet and soft in texture could not be mass-produced and sold because of shelf-life issues based on both sensory changes over time and food safety issues. The expansion of this area through both technology innovation and creative product design and development could bring new textures and flavors into the existing white spaces. Because 65% of the JP snacks evaluated were savory, the potential opportunity to create sweet or sweet and savory snacks is great.

Step 3: Opportunity Definition (Distinct, Early Features, Requirements, and Product Specifications)

Another essential part of NPD is defining the project scope, target market, as well as product features, attributes, and specifications [18,99]. The PCA plots generated from the sensory profiling of snacks can be used as guidelines to frame the sensory profile of new concepts and the direction of potential new product definitions and specifications. Descriptive profiling provided essential elements of the existing snacks such as appearance (color), shape, flavor, and texture attributes (physical components). These key attributes and components can be manipulated in iterative or “structured ways” to come up with new product configurations [54,94]. For example, the attributes of the PC1 and PC2 contributed the most to explaining the total variability from a list of key texture and appearance attributes. The strengths of these texture attributes are measurable and manipulatable to predict and develop new product candidates. Since texture has been identified as an important function of snack foods from which derive consumer desired benefits [64,100], and serves as the base of many snack food development projects, knowing those existing attributes is key information, both to provide reference points for “me-too” products and for companies interested in finding new opportunities. The descriptive analysis helped to quantify product attributes and translate them into measurable product characteristics [55].

The white spaces between snack groups identified by their texture attributes represent the gaps where new prototypes can be placed. The existing snacks’ (near to white space) key sensory specifications could be used as a starting point for prototype development. Developers can tweak the key sensory texture intensities by using consumer feedback. Sensory profiles of prototype products can be plotted on the same PCA plot to verify texture positioning. For example, there is a scarcity of snacks that are fibrous, cohesive, mealy, moist, and have a waxy mouthfeel for the JP market (Figure 3). Similarly, wide product space is available for snacks with other key sensory attributes such as firmness, chew count, and gritty.

For the SK snacks, large white spaces were found between and within each snack group (Figure 2). For example, the white space around Stacy’s pita original chips shows the unavailability of a similar product in the SK snack market. The developers can use the tested products as reference products to quantify texture specifications. Throughout the NPD process, the prototypes should be compared with the target product for the key attributes and other desirable sensory characteristics identified in descriptive profiling. The inclusion of either target or main competitive products makes it easier for developers to evaluate whether the newly developed prototypes adhere to the desired product concept [101].

Descriptive analysis is valuable for the replacement of essential components. A product developer can either replace essential components (for example, ingredient, flavors, or base material) of the product with something novel or close to the immediate background of the product that can accomplish the same necessary function. For example, the replacement of oil with plant sterols in mayonnaise. The plant sterols not only fulfill the functional requirement of providing structure and flavor carrying ability but also added health benefits by reducing serum cholesterol [24,102]. Once the desired product is fully developed, multiple consumer studies must be carried out to evaluate hedonics towards the newly developed product(s) and comparison must be made with current or competitive products. The foremost benefit of performing the descriptive analysis throughout the NPD is a detailed understanding of products. In addition, descriptive analysis is usually more cost-effective than consumer studies.

A product developer can also make several copies of an existing snack component and alter them in creative ways. For example, the development of purple corn tortilla chips on the line of regular yellow corn tortilla chips. Another creative way would be increasing the plant protein component of existing products for delivering more protein within existing product texture space. A smart developer can include several ideas (for example, environmentally sustainable ingredients, novel ingredients, plant proteins, less processing, and natural) to create niche product spaces but maintain similar texture profiles.

Step 4: Opportunity for Fine-Tuning (Iterative, Prototype Development, Test, Feedback, and Revise Iterations)

In rapidly changing consumer needs, it is not always possible to identify consumer needs and obtain correct product definitions. Developers should use iterative steps to build prototypes to fulfill identified white spaces. Sometimes, consumer requirements change in the time that passes between the beginning and end of development. Thus, the original product definition no longer satisfies consumer requirements.

Often consumers are not clear or fail to articulate what they need in the product until they see the product [103,104]. Thus, it is difficult to obtain an accurate product definition in the early stages of product development if the developer solely depends on explicit consumer inputs for idea generation. Because of limited exposure, consumer inputs are believed to restrict new ideas [26]. Instead, the product definition should be driven by presenting successive versions of the prototypes to consumers for feedback and verification. Iterative development is a dynamic process to capture accurate product definition by presenting a series of deliberative iterative prototypes to consumers. Therefore, the iterative development of prototypes is fluid, captures changing information, and floats the final products close to consumer requirements [18,26].

Information such as what consumers like or dislike and the value consumers see in prototypes should be gathered. The developer can revise, reset or plan the next (future) iteration about the benefits required, propositions, and product design based on gathered feedback.

Step 5: Opportunity Feasibility (Marketing, R&D, Engineering, Production)

Only those prototypes that address the needs of the consumer will be most likely to succeed and should be offered to product and other technology specialists to develop into a tangible product. Similarly, marketing must be involved to determine how products and market needs can be paired and promoted to produce successful launch and sales data.

### 4.1. Implications

In the current scenario of globalization and high competition, the methodology and results produced in this study could serve as a market-based source for innovation. The market-based approach is a form of open innovation that uses markets as a source for external knowledge [3]. The inclusion of this methodology in the innovation process can be beneficial for the industry. More specifically, it is beneficial for revenues from incremental innovation, reduced time to market, to achieve marginal improvements to existing products, and tremendously impact new product performance [3,105,106]. However, to capitalize market knowledge for innovation, food companies and developers are required to increase absorption capacity [107,108]. The adoption of this methodology could give developers an edge in the hyper competitive environment, capture merging and fast changing consumer needs, and other requirements that can be included in innovation process.

### 4.2. Limitations

The NPD methodology used in this study to generate new texture concepts could be used for other food product categories. However, researchers are advised to do rigorous homework before applying the suggested methodology to other markets or market categories. This study suggests the utility of sensory methods in market assessment and ideation. However, the adoption of these methods does not guarantee the success of new products, especially if the market has not been evaluated thoroughly. The prototypes developed by using this methodology only confront consumers with products developed within the existing framework of the market tested. This can be noted in two ways: (1) the products selected drive the PM and the descriptive profiling. If the products are not representative of the market or category tested, the results will have limited application or could even be misleading; (2) current products do not necessarily lead to “outside-the-box” innovation. It may be difficult to understand unfulfilled needs by examining the prototypes based on the existing marketplace. This is where the creativity of the product development team including the food scientists, sensory scientists, engineers, and marketing specialists need to come together to “imagine” and then create new product concepts and prototypes for testing.

Results projecting products from one market onto another also are not always successful depending on the similarity in preferences and consumer segments between countries. For example, one study showed that the same segments of consumers existed in multiple countries for a product (pomegranate juice) [109]. However, the proportion of consumers in those segments was completely different in the US and Spain, suggesting that a juice developed for the Spanish market may not be successful in the US. On the other hand, this could be the result of products not being readily available in certain countries or the difference in consumption rates among countries. Testing with consumers who regularly eat certain products in a category is quite different from testing with consumers who are new to the product type. Thus, prototypes developed using the JP and SK snack market framework could be a potential opportunity for the US market or maybe too far out of the current repertory of snack products to be successful. Testing with consumers for acceptance within the current framework and within an “altered framework” is required when testing completely new products.

### 4.3. Future Research

It is important to conduct comprehensive studies that analyze the impact of a market source innovation model on the performance of new products, incremental income and the cost associated with them. Although these are recommended methods, they need to be examined in a critical light based on case-studies and other use research. In addition, future studies must be conducted to explain what kinds of human capabilities the industry must develop and use to capitalize on external market knowledge.

## 5. Conclusions

The world is changing rapidly, i.e., more global, less predictable, and more abstruse. The product developers’ task is full of multi-faceted challenges. A plethora of literature has been published to deal with these challenges. For example, “open innovation, agile development, design thinking for ideation, stage-gate development, and lean product development”. The developers require more creative techniques than “just ask consumers what they want” to increase the chances of success in competitive markets.

This paper showed one method of how new product concepts can be developed using sensory science tools such as product categorization, PM, and descriptive profiling. This research approach for novel and distinctive market opportunities displays an innovative, practical side of NPD research as a compliment. This study also identified the foremost sensory attributes of the JP and SK snack foods that drive consumer benefits. The proposed methodology can be used by food manufacturers to develop new product ideas from unfamiliar markets.

The results of this study can help developers learn to find white spaces in the marketplace and fill these spaces by designing prototypes. The developers can use tested products (close to white spaces) for initial specifications and then build several concepts for consumer assessment. This study is unique in its approach because it allows developers to use sensory methods to put several new ideas on the table for refinement and consumer feedback. The significance of product innovation is critical to business prosperity and consumer satisfaction, however, the keys to success remain indefinable.

## Figures and Tables

**Table 1:** List of the Japan (JP) snacks screened for descriptive profiling.

Serial Numbers	Snacks	Manufacturer ^1^	PM Code * and Group Numbers **
1	3D corn bugle	-	23, group-2
2	Bourbon lubera rolls	Bourbon	13
3	Nagewa potato rings	Family Mart collection	4, group-5
4	Sesame wafer rolls	-	40, group-8
5	Seaweed coated crackers	-	34, group-6
6	Freeze-dried strawberries	Fukumi	16, group-4
7	Cheese-filled crackers	Family Mart collection	15, group-2
8	Plum meat snack	Seven Eleven	29, group-3
9	Baby star ramen	Oyatsu Company	33, group-6
10	Strawberry filled balls	Seven Eleven	25, group-4
11	Freeze-dried ice-cream cone	Glico	47, group-4
12	Cheese-filled rolls	Kirara	36, group-1
13	Squid chips	-	21, group-1
14	Unbranded rice crisps	-	41, group-6
15	Pasta shaped snack	Seven Eleven	1, group-1
16	Calbee Potato Sticks	Calbee	2
17	Pocky chocolate sticks	Glico	26, group-9
18	Pea crisps	Calbee	11, group-5
19	Sweet potato sticks	Family Mart collection	9, group-7
20	Unbranded seaweed crackers	-	35, group-1
21	Riska corn potage puffs	Riska	New
22	Bourbon rice crackers with cheese	Bourbon	New
23	Zaku curry filled snacks	-	New
24	Kameda nut clusters	Kameda	New
25	Renkon lotus root chips	Sokan group	New
26	Morianga bites	Morianga	New
27	Steamed plum seaweed	Family Mart Collection	New
28	Edamame crisps	Seven Eleven	New
29	Denroku crispy coated nuts	Denroku	New
30	Mayonnaise potato wedges	Seven Eleven	New
31	Soybean FL coated peanuts	Nuts.com	New
32	Peanut coated cotton candy balls	-	New
33	Lay’s classic potato chips	Frito-Lay	Anchor
34	Tostitos original corn chips	Frito-Lay	Anchor
35	Stacy’s pita original	Frito-Lay	Anchor

^1^ Products without a manufacturer listed are snacks either sold “on the street” or in local “snack” shops in packages without a label. * PM code is a 2-digit number used in the projective mapping plot as an identifier for snack samples. ** Group numbers are provided to identify the snack sample association in projective mapping grouping. New = a product added to this study; Anchor = a common U.S. product used for comparison purposes.

**Table 2:** List of South Korea (SK) snacks screened for descriptive profiling.

Serial Numbers	Snacks	Manufacturer ^1^	PM Code * and Group Numbers **
1	Orion turtle chips (Himalayan salt)	Orion	41, group-3
2	Orion Peanut Balls	Orion	44, group-4
3	Orion original potato chip	Orion	40
4	Taco chips	Lotte	28, group-1
5	Peanut crunchy bar	Koon brother	13, group-9
6	Pulmuone Crispy seaweed chips	Pulmuone	7, group-3
7	Dasang sweet potato sticks	Dasang	5, group-7
8	Soy sauce seaweed chips	Tempura Chips	60, group-2
9	Momali crown snack	Crown Co.	4, group-4
10	Haitai Rice Sticks	Haitai Calbee	52, group-1
11	Daiso orion potato chips	Orion	14
12	Florentin Coconut French dessert	Peacock	48, group-4
13	Heyroo Injeolmi snack	Heyroo	38, group-6
14	Heyroo Noodle snack	Heyroo	21, group-3
15	Heyroo sweet popcorn	Heyroo	43, group-4
16	Heyroo oranda clusters	Heyroo	46, group-5
17	Prawn snack	-	63, group-3
18	Laver Almond	Tom’s farm	31
19	Mushroom snack	-	15, group-6
20	Kims crispy roasted laver chips	Dongwon Yangban	57, group-7
21	Seed filled cookie	Lotte	New
22	Seaweed rolls	Only price 2000	New
23	Squid rice balls	-	New
24	Roasted lotus seeds	Daily super nuts	New
25	Baby crab crunch	Farm & Dale	New
26	Soft somjulmi snack	Peacock	New
27	Seaweed crisps	Cheiljedang	New
28	Honey butter cashew-nut	Tom’s Farm 1982	New
29	Yogurt cashew-nut	Murgerbon	New
30	Tofu snack	Hav’eat	New
31	NongHyup grain crisps	NongHyup	New
32	Chicken shaped snack	Lotte	New
33	Lay’s classic potato chips	Frito-Lay	Anchor
34	Stacy’s pita Original	Frito-Lay	Anchor
35	Tostitos original corn chips	Frito-Lay	Anchor

^1^ Products without a manufacturer listed are snacks either sold “on the street” or in local “snack” shops in packages without a label. * PM code is a 2-digit number used in the projective mapping plot as an identifier for snack samples. ** Group numbers are provided to identify the snack sample association in projective mapping grouping. New = a product added to this study; Anchor = a common U.S. product used for comparison purposes.

**Table 3:** Group identified in the projective mapping of the JP snacks. Group number, number of snacks in each group, texture, snack type, flavor, and snack names.

Groups	Number of Snacks	Texture and Flavor	Snacks Type and Flavor	Examples Snacks Names
Group-1	14	Moderate hard bite texture with mild to a strong savory flavor	Type—crackers, wafers, rolls, puffs Flavor—cheese, squid, savory	Ramen noodle snack, shrimp chips, seaweed crackers, squid snack, rice crackers, coated rice crackers, rice crackers, pasta shape fried snack, ginseng root chips, cheese-filled rolls
Group-2	6	Soft bite texture with a low savory flavor	Type—crackers, wafers, rolls, puffs Flavor—cheese, sweet, sesame	3D corn bugles, corn puffs, squid crackers, shrimp crackers, cheese-filled sticks, unbranded rice crackers
Group-3	5	Extremely soft-chewy with a strong savory flavor	Type—seafood and meat Flavor—seafood, fish	Dried squid, plum meat, dried fish, cheese with cod, spicy grilled kamaboko fish
Group-4	5	Extremely soft with a strong sweet flavor	Type—cake, freeze-dried, puffed balls Flavor—strawberry, chocolate, sweet	Baumkuchen cake, freeze-dried strawberries, strawberry filled puffed balls, freeze-dried strawberry ice-cream cone, chocolate sweet treats
Group-5	5	Moderate hard bite with a bland taste	Type—sticks, chips, crisps Flavor—bland, plain, salt	Fried rice crackers, potato rings, pea sticks, rice crackers, fried rice crackers with peanuts
Group-6	4	Extremely hard bite with a very strong savory flavor	Type—hard grain crackers Flavor—seaweed	Seaweed crackers, baby star ramen noodle snack, unbranded fried snack, unbranded crackers
Group-7	3	Extremely hard bite with a strong sweet flavor	Type—sticks, crackers Flavor -sweet	Unbranded baked crackers, soybean coated walnuts, sweet potato sticks
Group-8	3	Moderate hard bite with a mildly sweet flavor	Type—puffs, crackers, Flavor—sweet, chocolate	Chocolate-coated baked rice puffs, sesame wafer rolls, sugar granules coated crackers
Group-9	2	Moderate soft bite with a mildly sweet flavor	Type—sticks Flavor—sweet, chocolate, sesame	Rice crackers with sesame seeds, Pocky chocolate sticks

**Table 4:** Group identified in the projective mapping of the SK snacks. Group number, number of snacks in each group, snack type, texture, flavor, and snack names.

Groups	Number of Snacks	Texture and Flavor	Snacks Type and Flavor	Examples Snacks Names
Group-1	4	Extremely hard bite with an extremely strong savory flavor	Type—chips, sticks Flavor—savory, corn, garlic, seaweed	Binggre smoky bacon chip with spicy beef flavor, Brito’s snacks, Mexican taco chip, Mister free’d chia seed tortilla chips, Haitai spicy Rice cake sticks
Group-2	6	Moderate hard bite with a mild savory flavor	Type—potato chips, fish chips Flavor—seaweed, chicken, crab, savory	Nong shim cuttlefish roasted butter chips, crab-shaped baked snack, Nongshim chicken leg snack, Nongshim potato chips, Pulmuone seaweed chips
Group-3	10	Moderate hard bite with a bland or little sweet flavor	Type—chips, trail mix, crackers Flavor—soy, seaweed, bland, salt	Orion turtle chips, Peacock Seoul crispy rice chips, Pulmuone crispy seaweed snack, Soy Sauce Tempura Seaweed Snacks, Heyroo noodle snack, Prawn snack, Heyroo seaweed tofu snack, dried fish snack, Mum Mum rice rusks, ChungWoo Fermented Hardtack crackers, The Kims crispy laver chips
Group-4	7	Moderate hard bite with a mildly sweet flavor	Type—nuts, chips, crackers, Flavor—squid, coffee, sweet	Orion squid flavored peanut balls, crunchy and tasty deep anchovy fried, Momali shinchon (crown) snack, Peacock Florentin coconut French dessert, Heyroo sweet popcorn kernel covered with sweet butter scent, coffee coated peanut, Nobrand coconut sticks
Group-5	4	Extremely hard bite with an extremely sweet flavor	Type—puffs, chips Flavor—sweet, peanut	Nobrand seashell-shaped snack, Haitai matdongsan peanut crunch, Heyroo oranda snacks, Amigo chips
Group-6	11	Slight hard bite with a mildly sweet flavor	Type—chips, sticks, crisps, crackers, rolls Flavor—sweet, rice, seaweed, fish	Fried butter potato chips, Orion potato sticks, Orion Gosomi Sweet Cookie Cracker, heyroo injeolmi traditional Rice Cake Snack Crispy Coated by Bean powder, Heyroo egg snacks, Shinhwa seasoned dried fish meat, Haitai calbee sweet potato chips, Crown rice crackers, Market O nature mushroom snack, Big roll grilled seaweed roll: classic flavor, Pulmuone snack chip
Group-7	6	Slight soft bite with a bland or little sweet flavor	Type—sticks, chips, crisps Flavor—seaweed, sweet, sesame	Roasted sweet potato chew snack with pineapple flavor, coconut seaweed baby snack, seaweed snack with white sesame, The Kims crispy roasted laver chips, Team Korea crispy laver snack, K-fish seaweed chips
Group-8	3	Very soft chewy texture with a mild savory flavor	Type—Jerky, dried meat Flavor—seafood and meat	Roast horse mackerel, baked cheese dried squid, hot pork jerky
Group-9	3	Very soft texture with a mild to very sweet flavor	Type—grain bars, crisps Flavor—sweet, banana	Mybizcuit peanut crunchy bar, Premium Grain bars, Kiddylicious banana crispy

## Supplementary Materials

The following are available online at https://www.mdpi.com/2304-8158/10/2/474/s1, Table S1: Definitions and references.

## References

[1] Fuller G.W. (2016). New Food Product Development.

[2] Guiné R.P.F., Ramalhosa E.C.D., Valente L.P. (2016). New Foods, New Consumers: Innovation in Food Product Development. Curr. Nutr. Food Sci..

[3] Santoro G., Vrontis D., Pastore A. (2017). External knowledge sourcing and new product development. Br. Food J..

[4] Bresciani S. (2017). Open, networked and dynamic innovation in the food and beverage industry. Br. Food J..

[5] Della Corte V., Del Gaudio G., Sepe F. (2018). Innovation and tradition-based firms: A multiple case study in the agro-food sector. Br. Food J..

[6] Merieux NutriSciences, Lascom (2018). How to Facilitate Your Product Development in a Global Regulatory Environment. A white paper. Food and Beverage. http://www.lascom.com/wp-content/uploads/2018/05/White-Paper_Merieux-NutriSciences-Lascom_How-to-facilitate-product-development-in-a-global-regulatory-environment.pdf.

[7] Stewart-Knox B., Parr H., Bunting B., Mitchell P. (2003). A model for reduced fat food product development success. Food Qual. Prefer..

[8] Banović M., Krystallis A., Guerrero L., Reinders M.J. (2016). Consumers as co-creators of new product ideas: An application of projective and creative research techniques. Food Res. Int..

[9] De Pelsmaeker S., Gellynck X., Delbaere C., Declercq N., Dewettinck K. (2015). Consumer-driven product development and improvement combined with sensory analysis: A case-study for European filled chocolates. Food Qual. Prefer..

[10] Murley T., Kumar R., Chambers E., Chambers D., Ciccone M., Yang G. (2020). A process for evaluating a product category in an unfamiliar country: Issues and solutions in a case study of snacks in Japan. J. Sens. Stud..

[11] Horvat A., Granato G., Fogliano V., Luning P.A. (2019). Understanding consumer data use in new product development and the product life cycle in European food firms—An empirical study. Food Qual. Prefer..

[12] Johnson M.W. (2018). Reinvent Your Business Model: How to Seize the White Space for Transformative Growth.

[13] Jagtap S., Duong L.N.K. (2019). Improving the new product development using big data: A case study of a food company. Br. Food J..

[14] Dijksterhuis G. (2016). New product failure: Five potential sources discussed. Trends Food Sci. Technol..

[15] Nielson (2014). Breakthrough innovation report. https://www.nielsen.com/us/en/insights/report/2014/breakthrough-innovation-report/.

[16] Kemp S., Hort J. (2015). Trends in sensory science. Food Sci. Technol..

[17] Asioli D., Varela P., Hersleth M., Almli V.L., Olsen N.V., Næs T. (2017). A discussion of recent methodologies for combining sensory and extrinsic product properties in consumer studies. Food Qual. Prefer..

[18] Cooper R.G. (2019). The drivers of success in new-product development. Ind. Mark. Manag..

[19] Costa A.I.A., Jongen W.M.F. (2006). New insights into consumer-led food product development. Trends Food Sci. Technol..

[20] Grujić S., Odžaković B., Ciganović M. Sensory analysis as a tool in the new food product development. Proceedings of the II International Congress Food Technology Quality and Safety.

[21] Ryynänen T., Hakatie A. (2014). “We must have the wrong consumers”—A case study on new food product development failure. Br. Food J..

[22] Simeone M., Marotta G. (2010). Towards an integration of sensory research and marketing in new food products development: A theoretical and methodological review. Afr. J. Bus. Manag..

[23] Talavera M., Chambers E. (2017). Using sensory sciences help products succeed. Br. Food J..

[24] MacFie H.J.H. (2007). Index. Consumer-Led Food Product Development.

[25] Crofton E.C., Scannell A.G.M. (2020). Snack foods from brewing waste: Consumer-led approach to developing sustainable snack options. Br. Food J..

[26] Cuny C., Petit C., Allain G. (2020). Capturing implicit texture-flavour associations to predict consumers’ new product preferences. J. Retail. Consum. Serv..

[27] Godin L., Laakso S., Sahakian M. (2020). Doing laundry in consumption corridors: Wellbeing and everyday life. Sustain. Sci. Pract. Policy.

[28] Mahama-Musah F., Vanhaverbeke L., Gillet A. (2020). The impact of personal, market- and product-relevant factors on patronage behaviour in the automobile tyre replacement market. J. Retail. Consum. Serv..

[29] Mora M., Romeo-Arroyo E., Torán-Pereg P., Chaya C., Vázquez-Araújo L. (2020). Sustainable and health claims vs sensory properties: Consumers’ opinions and choices using a vegetable dip as example product. Food Res. Int..

[30] Zocchi D.M., Piochi M., Cabrino G., Fontefrancesco M.F., Torri L. (2020). Linking producers’ and consumers’ perceptions in the valorisation of non-timber forest products: An analysis of Ogiek forest honey. Food Res. Int..

[31] Donelan A.K., Chambers D.H., Chambers E., Godwin S.L., Cates S.C. (2016). Consumer Poultry Handling Behavior in the Grocery Store and In-Home Storage. J. Food Prot..

[32] Pawera L., Khomsan A., Zuhud E.A.M., Hunter D., Ickowitz A., Polesny Z. (2020). Wild food plants and trends in their use: From knowledge and perceptions to drivers of change in West Sumatra, Indonesia. Foods.

[33] Talavera M., Sasse A.M. (2019). Gathering consumer terminology using focus groups—An example with beauty care. J. Sens. Stud..

[34] Hoppu U., Puputti S., Mattila S., Puurtinen M., Sandell M. (2020). Food consumption and emotions at a salad lunch buffet in a multisensory environment. Foods.

[35] Bryant C., van Nek L., Rolland N.C.M. (2020). European markets for cultured meat: A comparison of germany and france. Foods.

[36] Chambers E., Tran T., Chambers E. (2019). Natural: A $75 billion word with no definition—Why not?. J. Sens. Stud..

[37] Murley T., Chambers E. (2019). The influence of colorants, flavorants and product identity on perceptions of naturalness. Foods.

[38] Doungtip P., Sriwattana S., Kim K.T. (2020). Understanding Thai consumer attitudes and expectations of ginseng food products. J. Sens. Stud..

[39] Aguiar L.A., Melo L., de Lacerda de Oliveira L. (2019). Validation of rapid descriptive sensory methods against conventional descriptive analyses: A systematic review. Crit. Rev. Food Sci. Nutr..

[40] Cartier R., Rytz A., Lecomte A., Poblete F., Krystlik J., Belin E., Martin N. (2006). Sorting procedure as an alternative to quantitative descriptive analysis to obtain a product sensory map. Food Qual. Prefer..

[41] Pagès J., Cadoret M., Lê S. (2010). The sorted napping: A new holistic approach in sensory evaluation. J. Sens. Stud..

[42] Risvik E., McEwan J.A., Rødbotten M. (1997). Evaluation of sensory profiling and projective mapping data. Food Qual. Prefer..

[43] Zhang T., Lusk K., Mirosa M., Oey I. (2016). Understanding young immigrant Chinese consumers’ freshness perceptions of orange juices: A study based on concept evaluation. Food Qual. Prefer..

[44] He W., Chung H.Y. (2019). Comparison between quantitative descriptive analysis and flash profile in profiling the sensory properties of commercial red sufu (Chinese fermented soybean curd). J. Sci. Food Agric..

[45] Brand J., Kidd M., van Antwerpen L., Valentin D., Næs T., Nieuwoudt H.H. (2018). Sorting in combination with quality scoring: A tool for industry professionals to identify drivers of wine quality rapidly. S. Afr. J. Enol. Vitic..

[46] González-Mohíno A., Antequera T., Pérez-Palacios T., Ventanas S. (2019). Napping combined with ultra-flash profile (UFP) methodology for sensory assessment of cod and pork subjected to different cooking methods and conditions. Eur. Food Res. Technol..

[47] Cliceri D., Dinnella C., Depezay L., Morizet D., Giboreau A., Appleton K.M., Hartwell H., Monteleone E. (2017). Exploring salient dimensions in a free sorting task: A cross-country study within the elderly population. Food Qual. Prefer..

[48] Saldaña E., Martins M.M., Behrens J.H., Valentin D., Selani M.M., Contreras-Castillo C.J. (2020). Looking at non-sensory factors underlying consumers’ perception of smoked bacon. Meat Sci..

[49] Soares E.K.B., Esmerino E.A., Ferreira M.V.S., da Silva M.A.A.P., Freitas M.Q., Cruz A.G. (2017). What are the cultural effects on consumers’ perceptions? A case study covering coalho cheese in the Brazilian northeast and southeast area using word association. Food Res. Int..

[50] Katiyo W., Coorey R., Buys E.M., Kock H.L. (2020). Consumers’ perceptions of intrinsic and extrinsic attributes as indicators of safety and quality of chicken meat: Actionable information for public health authorities and the chicken industry. J. Food Sci..

[51] Thomas S., Chambault M. (2016). Integrating the Packaging and Product Experience in Food and Beverages.

[52] Mayhew E., Schmidt S., Lee S.Y. (2016). Napping—Ultra flash profile as a tool for category identification and subsequent model system formulation of caramel corn products. J. Food Sci..

[53] Valentin D., Cholet S., Nestrud M., Abdi H., Kemp S.E., Hort J., Hollowood T. (2018). Projective mapping and sorting tasks, Chapter 15. Descriptive Analysis in Sensory Evaluation.

[54] Lawless H.T., Heymann H. (2013). Sensory Evaluation of Food.

[55] Chambers E., Kemp S., Hort J., Hollowood T. (2018). Consensus Methods for Descriptive Analysis. Descriptive Analysis in Sensory Evaluation.

[56] Chambers E. (2019). Analysis of sensory properties in foods: A special issue. Foods.

[57] Luchsinger S.E., Kropf D.H., García Zepeda C.M., Chambers IV E., Hollingsworth M.E., Hunt M.C., Marsden J.L., Kastner C.L., Kuecker W.G. (1996). Sensory analysis and consumer acceptance of irradiated boneless pork chops. J. Food Sci..

[58] Muñoz A.M., Chambers E.I. (1993). Relating measurements of sensory properties to consumer acceptance of meat products. Food Technol..

[59] Suwonsichon S. (2019). The importance of sensory lexicons for research and development of food products. Foods.

[60] Yang J., Lee J. (2019). Application of sensory descriptive analysis and consumer studies to investigate traditional and authentic foods: A review. Foods.

[61] Van Kleef E., van Trijp H.C.M., Luning P. (2005). Consumer research in the early stages of new product development: A critical review of methods and techniques. Food Qual. Prefer..

[62] Tran T., James M.N., Chambers D., Koppel K., Chambers E. (2019). Lexicon development for the sensory description of rye bread. J. Sens. Stud..

[63] Chun S., Chambers E., Han I. (2020). Development of a Sensory Flavor Lexicon for Mushrooms and Subsequent Characterization of Fresh and Dried Mushrooms. Foods.

[64] Kumar R., Chambers E. (2019). Lexicon for multiparameter texture assessment of snack and snack-like foods in English, Spanish, Chinese, and Hindi. J. Sens. Stud..

[65] Sharma C., Chambers E., Jayanty S.S., Sathuvalli Rajakalyan V., Holm D.G., Talavera M. (2020). Development of a lexicon to describe the sensory characteristics of a wide variety of potato cultivars. J. Sens. Stud..

[66] de Godoy R.C.B., Chambers E., Yang G. (2020). Development of a preliminary sensory lexicon for mate tea. J. Sens. Stud..

[67] Laird H., Miller R.K., Kerth C.R., Chambers E. (2017). The Flavor and Texture Attributes of Ground Beef. Flavor Texture Attrib. Gr. Beef.

[68] Jaffe T.R., Wang H., Chambers E. (2017). Determination of a lexicon for the sensory flavor attributes of smoked food products. J. Sens. Stud..

[69] Culbert J.A., Ristic R., Ovington L.A., Saliba A.J., Wilkinson K.L. (2017). Influence of production method on the sensory profile and consumer acceptance of Australian sparkling white wine styles. Aust. J. Grape Wine Res..

[70] Lee J., Chambers E., Chambers D.H., Chun S.S., Oupadissakoon C., Johnson D.E. (2010). Consumer acceptance for green tea by consumers in the United States, Korea and Thailand. J. Sens. Stud..

[71] Bowen A.J., Blake A., Tureček J., Amyotte B. (2019). External preference mapping: A guide for a consumer-driven approach to apple breeding. J. Sens. Stud..

[72] Sharma C., Jayanty S.S., Chambers E., Talavera M. (2020). Segmentation of potato consumers based on sensory and attitudinal aspects. Foods.

[73] Salvador A., Varela P., Sanz T., Fiszman S.M. (2009). Understanding potato chips crispy texture by simultaneous fracture and acoustic measurements, and sensory analysis. LWT Food Sci. Technol..

[74] Antmann G., Ares G., Salvador A., Varela P., Fiszman S.M. (2011). Exploring and explaining creaminess perception: Consumers’ underlying concepts. J. Sens. Stud..

[75] Frøst M.B., Janhøj T. (2007). Understanding creaminess. Int. Dairy J..

[76] De Pelsmaeker S., Dewettinck K., Gellynck X. (2013). The possibility of using tasting as a presentation method for sensory stimuli in conjoint analysis. Trends Food Sci. Technol..

[77] Adriana L.S., Mauricio C., Delores C., Loreida T., Kadri K., Edgar C., Yu H. (2019). Benefits, Challenges, and Opportunities of Conducting a Collaborative Research Course in an International University Partnership: A Study Case Between Kansas State University and Tallinn University of Technology. J. Food Sci. Educ..

[78] Corley J. (2017). Finding the white space in natural personal care: How Olivina identified a niche (natural male grooming) and fast-tracked from formulation to shelf in nine months. Glob. Cosmet. Ind..

[79] Thompson A. (2019). Kinder Joy, M&M’S Caramel top IRI’s list of 2018 New Product Pacesetters. Candy Ind..

[80] Phan U.T.X., Chambers E. (2016). Application of An Eating Motivation Survey to Study Eating Occasions. J. Sens. Stud..

[81] Pagès J. (2005). Collection and analysis of perceived product inter-distances using multiple factor analysis: Application to the study of 10 white wines from the Loire Valley. Food Qual. Prefer..

[82] Chambers E., Chambers D.H., Bleibaum R. (2020). Chapter 1 | Consensus Profile Methods Derived from the Flavor Profile Method. Descriptive Analysis Testing for Sensory Evaluation.

[83] Griffin L.E., Dean L.L., Drake M.A. (2017). The development of a lexicon for cashew nuts. J. Sens. Stud..

[84] Chambers E., Bowers J.A., Dayton A.D. (1981). Statistical designs and panel training/experience for sensory analysis. J. Food Sci..

[85] Chambers E., Smith E.A. (1993). Effects of testing experience on performance of trained sensory panelists. J. Sens. Stud..

[86] Chambers D.H., Allison A.M.A., Chambers IV E. (2004). Training effects on performance of descriptive panelists. J. Sens. Stud..

[87] Otremba M.M., Dikeman M.E., Milliken G.A., Stroda S.L., Unruh J.A., Chambers IV E. (1999). Interrelationships among evaluations of beef longissimus and semitendinosus muscle tenderness by Warner-Bratzler shear force, a descriptive-texture profile sensory panel, and a descriptive attribute sensory panel. J. Anim. Sci..

[88] Belisle C., Adhikari K., Chavez D., Phan U.T.X. (2017). Development of a lexicon for flavor and texture of fresh peach cultivars. J. Sens. Stud..

[89] Chambers E., Lee J., Chun S., Miller A.E. (2012). Development of a Lexicon for Commercially Available Cabbage (Baechu) Kimchi. J. Sens. Stud..

[90] Muñoz A.M., Chambers IV E., Hummer S. (1996). A multifaceted category research study: How to understand a product category and its consumer responses. J. Sens. Stud..

[91] Suwonsichon S., Chambers Iv E., Kongpensook V., Oupadissakoon C. (2012). Sensory lexicon for mango as affected by cultivars and stages of ripeness. J. Sens. Stud..

[92] Thompson K.R., Chambers D.H., Chambers IV E. (2009). Sensory characteristics of ice cream produced in the U.S.A. and ITALY. J. Sens. Stud..

[93] Grunert K.G. (2017). Consumer Trends and New Product Opportunities in the Food Sector.

[94] Simms C., Trott P. Packaging Dependent Products: How do Firms in the Packaged Food Sector Manage the Development of new Packaging Opportunities?. Proceedings of the 12th European Conference on Innovation and Entrepreneurship, Academic Conferences International Limited.

[95] Wind J., Mahajan V. (1997). Issues and Opportunities in New Product Development: An Introduction to the Special Issue. J. Mark. Res..

[96] Cooper R.G. (2018). Best practices and success drivers in new product development. Handbook of Research on New Product Development.

[97] Costa G.M., Paula M.M., Costa G.N., Esmerino E.A., Silva R., Freitas M.Q., Barão C.E., Cruz A.G., Pimentel T.C. (2020). Preferred attribute elicitation methodology compared to conventional descriptive analysis: A study using probiotic yogurt sweetened with xylitol and added with prebiotic components. J. Sens. Stud..

[98] Moussaoui K.A., Varela P. (2010). Exploring consumer product profiling techniques and their linkage to a quantitative descriptive analysis. Food Qual. Prefer..

[99] Cooper R.G. (2017). Winning at New Products: Creating Value through Innovation.

[100] Kumar R., Chambers E. (2019). Understanding the terminology for snack foods and their texture by consumers in four languages: A qualitative study. Foods.

[101] O’sullivan M. (2017). Index. A Handbook for Sensory and Consumer-Driven New Product Development.

[102] Goldenberg J., Mazursky D. (2002). Creativity in Product Innovation.

[103] Reid S.E., de Brentani U. (2004). The Fuzzy Front End of New Product Development for Discontinuous Innovations: A Theoretical Model. J. Prod. Innov. Manag..

[104] Savela-Huovinen U., Muukkonen H., Toom A. (2018). Sensory expert assessor’s learning practices at workplace: Competencies and contexts in sensory evaluation. J. Sens. Stud..

[105] Knudsen M.P. (2007). The relative importance of interfirm relationships and knowledge transfer for new product development success. J. Prod. Innov. Manag..

[106] Capitanio F., Coppola A., Pascucci S. (2010). Product and process innovation in the Italian food industry. Agribusiness.

[107] Chen Y., Vanhaverbeke W., Du J. (2016). The interaction between internal R&D and different types of external knowledge sourcing: An empirical study of Chinese innovative firms. R D Manag..

[108] Zobel A.K. (2017). Benefiting from Open Innovation: A Multidimensional Model of Absorptive Capacity. J. Prod. Innov. Manag..

[109] Koppel K., Chambers E., Vázquez-Araújo L., Timberg L., Carbonell-Barrachina T.A., Suwonsichon S. (2014). Cross-country comparison of pomegranate juice acceptance in Estonia, Spain, Thailand, and United States. Food Qual. Prefer..

