# Supplementary material for: Generating New Snack Food Texture Ideas Using Sensory and Consumer Research Tools: A Case Study of the Japanese and South Korean Snack Food Markets
Source: Foods. 2021 Feb 22;10(2):474. doi: 10.3390/foods10020474 (PMC7927123; doi:10.3390/foods10020474)
Supplement: Supplementary file 1 [file foods-10-00474-s001.pdf]

Table S1. Definitions and references.

	**Appearance**	
Evenness of color:	The perceived uniformity of color ranked from uneven = 0.0 to even = 15.0.	
	Reference:	Saltines = 2.5
		Pepperidge Farm Bordeaux Cookies = 9.0
Oily:	Impression of oil present on the surface of the sample.	
	Reference:	Ritz Cracker = 2.5
		Planters Cocktail Peanuts = 6.0
Particles:	The appearance of small pieces relatively harder than surrounding product.	
	Reference:	Hunt's Snack Pack Tapioca Pudding = 2.0
		Kozy Shack Rice Pudding = 8.0
Porous:	Presence of pores/air bubbles inside the mass.	
	Reference:	Cheerios = 8.0
Powdery:	Perception of a powdery substance/ coating on the product.	
	Reference:	Fritos corn chips = 4.0
		Cheetos cheese Puff = 7.0
Puffiness:	The degree to which air has been used to create an expanded, less dense appearing product.	
	Reference:	Cheetos Crunchy = 5.0
		Cheetos cheese Puff = 12.0
Surface shine:	The amount of gloss or shine on the surface of the product.	
	Reference:	Hiland’s Sour cream = 10.0
Surface roughness:	Indentations/bumps on surface; smooth to rough.	
	Reference:	Cheerios = 5.0
		Wheaties = 9.0
Uniformity of surface:	Perceived degree of evenness of top of product. The absence of valleys, raised areas, cracks, blisters, etc.	
	Reference:	Thomas Plain bagel (top) = 8.0
Wet/moist appearance	The visual perception of product moistness. (Dry to moist).	
	Reference:	Malt-O-Meal = 6.0
		Bush’s Pinto beans (canned) = 12.0
	**Texture**	
Adhesive:	The degree to which the product sticks to the mouth surfaces during mastication. None to much.	
	Reference:	Musselman's Apple Butter = 3.5
		Planters Cocktail Peanuts = 8.5
Chalky mouthfeel:	Chalky: a perception of a thin, even powdery coating on the tongue and mouth surface during mastication.	
	Reference:	Philadelphia Cream Cheese Block = 5.0
		Eagle brand sweetened condensed milk = 8.5
Chew count:	Number of chews required to hydrate sample and bring to a state ready to swallow. The sample is chewed on one side of the mouth only.	
	Reference:	Thomas English Muffin = 7.5
Cohesiveness:	The degree to which the sample deforms prior to breaking apart when compressed once between the molar teeth (least to most).	
	Reference:	Wheaties = 4.0

		Cheerios = 7.0
		Kellogg's Rice Krispies Treat= 10.0
Cohesiveness of mass:	Rinse mouth with water or drink a sip of water. The degree to which the mass holds together during mastication after 5–7 chews.	
	Reference:	Wheaties= 2.5
		Sara Lee Butter Pound Cake= 9.0
Dissolvability:	Rate and degree to which product dissolves in the mouth during mastication.	
	Reference:	Kroger mozzarella Cheese = 2.0
		Kroger fat free original cream cheese = 7.5
		Gerber 1st foods apple sauce = 13.0
Doughy:	A moist, under baked impression associated with grain product.	
	Reference:	Wonder Bread = 4.5
Fibrous:	The perception of filaments or strands of muscle tissue or plant fiber.	
	Reference:	Chicken Breast= 7.5
Firmness:	The force required to bite completely through the sample with the molar teeth, evaluated on first bite down with the molars.	
	Reference:	Kraft American cheese singles = 4.0
		Thomas English Muffin= 7.5
Fracturability:	The force with which the sample ruptures. Evaluate on first bite down with the molars.	
	Reference:	Cheerios = 4.0
		Fritos corn chips = 10.0
Gritty:	The perception of small, hard, sharp particles reminiscent of sand, or granules in pears.	
	Reference:	Malt-O-Meal = 2.0
		Jiffy Corn Bread Mix = 5.0
Initial crispness:	The intensity of audible noise at first bite with molars. One Cheerio, 2 Wheaties.	
	Reference:	Cheerios = 4.5
		Wheaties = 10.5
Mealy:	The perception of fine, soft, somewhat rounded smooth particles very evenly distributed within the product itself. Perceived as the product is broken down during mastication but is a geometrical attribute within the product and is not created by the mastication.	
	Reference:	Kroger/Musselman's Applesauce chunky = 5.0
Moistness:	The perceived amount of moisture in the product (dry to wet).	
	Reference:	Triscuit crackers = 1.5
		Kraft Mild cheedar cheese = 5.0
Oily mouthfeel:	The sensation of oily coating on mouth surface during mastication.	
	Reference:	Kraft Cool whip = 6.0
		Fritos corn chips = 9.0
Particle size:	Perception of the size of particles within the sample from small to large. During mastication, after five chews, pressing the tongue against roof of mouth. Place 2 cheerios in the mouth. Place two smaller sized Wheaties or one large.	
	Reference:	Cheerios = 3.0
		Wheaties = 6.0

Particles/residuals:	The amount of small pieces of sample remaining in mouth just after swallowing. This does not incorporate tooth packing and refers only to particulate matter on mouth surfaces other that in and between the molar teeth. This may include fibers, flakes or granules.	
	Reference:	Cheerios = 3.0
		Wheaties = 7.0
Roughness of mass:	The degree of abrasiveness of particles perceived when gently manipulating the mass against the palate after 5–7 chews.	
	Reference:	Cheerios = 4.0
		Wheaties = 8.0
		Triscuit crackers = 11.0
Roughness of surface:	The amount of indentations/bumps and surface abrasions which can be perceived by gently manipulating one piece between the lips.	
	Reference:	Lay's Original Potato Chip = 5.0
		Pepperidge Farm Bordeaux cookies = 7.0
		Triscuit crackers = 11.0
Sustained crispness:	The perceived duration of crispiness (audible noise) maintained that is equal to the first bite crispness.	
	Reference:	Cheerios = 4.0
Sustained fracturability:	The duration of force with which the sample ruptures that is equal to the first bite.	
	Reference:	Cheerios = 3.0
		Wheaties = 6.0
Tooth packing:	The amount of sample packed in a between the molar teeth after swallowing.	
	Reference:	Cheerios = 3.5
		Wheaties = 7.0
Uniformity of bite:	Degree to which the product changes from start to finish in the first bite with the molars. If the force necessary to bite through the sample changes during the bite, the product is non-uniform. The more consistent the force, the more it is uniform.	
	Reference:	Twix Candy Bar = 5.0 (less uniform)
		Kellogg's Rice Krispies Treat = 10.0 (more uniform)
Waxy mouthfeel:	The sensation of waxy coating on the mouth surface during mastication.	
	Reference:	Hershey's Butterscotch Chips = 8.5
